# Cognitive Benefits of Physical Exercise, Physical–Cognitive Training, and Technology-Based Intervention in Obese Individuals with and without Postmenopausal Condition: A Narrative Review

**DOI:** 10.3390/ijerph192013364

**Published:** 2022-10-16

**Authors:** Puntarik Keawtep, Wanachaporn Wichayanrat, Sirinun Boripuntakul, Siriporn C. Chattipakorn, Somporn Sungkarat

**Affiliations:** 1 Department of Physical Therapy, Faculty of Associated Medical Sciences, Chiang Mai University, Chiang Mai 50200, Thailand; 2 Research Group of Modern Management and Information Technology, College of Arts, Media and Technology, Chiang Mai University, Chiang Mai 50200, Thailand; 3 Neurophysiology Unit, Cardiac Electrophysiology Research and Training Center, Faculty of Medicine, Chiang Mai University, Chiang Mai 50200, Thailand; 4 Department of Oral Biology and Diagnostic Sciences, Faculty of Dentistry, Chiang Mai University, Chiang Mai 50200, Thailand

**Keywords:** exercise, physical activity, physical–cognitive training, obesity, menopause, cognitive function

## Abstract

Obesity and estrogen deprivation have been identified as significant risk factors for cognitive impairment. Thus, postmenopausal conditions when paired with obesity may amplify the risks of developing dementia. Physical exercise has been recommended as a primary treatment for preventing obesity-related comorbidities and alleviating menopausal symptoms. This narrative review aimed to summarize the effects of exercise on cognition in obese individuals with and without menopausal condition, along with potential physiological mechanisms linking these interventions to cognitive improvement. Research evidence has demonstrated that exercise benefits not only physical but also cognitive and brain health. Among various types of exercise, recent studies have suggested that combined physical–cognitive exercise may exert larger gains in cognitive benefits than physical or cognitive exercise alone. Despite the scarcity of studies investigating the effects of physical and combined physical–cognitive exercise in obese individuals, especially those with menopausal condition, existing evidence has shown promising findings. Applying these exercises through technology-based interventions may be a viable approach to increase accessibility and adherence to the intervention. More evidence from randomized clinical trials with large samples and rigorous methodology is required. Further, investigations of biochemical and physiological outcomes along with behavioral changes will provide insight into underlying mechanisms linking these interventions to cognitive improvement.

## 1. Introduction

The prevalence of obesity in all age groups, from the pediatric to geriatric population, is growing worldwide [1,2]. It has been well established that obesity is a major risk factor for several non-communicable diseases such as diabetes mellitus and cardiovascular disease [3,4]. The European Society of Endocrinology Clinical Practice Guideline and The Obesity Society state that obesity exacerbates the age-related decline in physical function and leads to frailty [5,6]. Obesity has negative impacts not only on metabolic and physical function but also on cognitive function [7,8,9]. The incidence rates of cognitive decline are more common in women compared to men [10]. A previous study found that the lifetime risk of dementia and Alzheimer’s disease in midlife women was twice that of men [11]. Obesity, particularly in midlife, is critical, as it has been linked to an increased risk of developing dementia and Alzheimer’s disease in later life [7,8]. A growing body of evidence has revealed that obesity is associated with the reduction of global cognitive function [12] and specific cognitive domains including executive function [12,13], memory [14], attention [12], and language [14]. Several animal and clinical studies demonstrated that obesity is linked with pathophysiological changes (e.g., metabolic dysfunction, insulin resistance, leptin resistance, systemic inflammation, and adiponectin dysregulation), which result in the reduction of neurotrophic factors, neurogenesis, angiogenesis, and consequently lead to cognitive impairment [12,13,15,16,17]. Higher body mass index (BMI) was related to a greater risk of dementia when BMI was measured more than 20 years before dementia diagnosis (during midlife), but lower BMI predicted dementia when BMI was measured closer to dementia diagnosis (in later life) [18].

The reduction of estrogen due to menopause is also associated with cognitive impairment [7,8,16]. A meta-analysis study reported that women who underwent the postmenopausal period had poorer cognitive performance than pre- and perimenopausal periods [19]. Given positive influences of estrogen on the brain (e.g., inducing spinogenesis and synaptogenesis in the prefrontal cortex and hippocampus), estrogen deprivation could lead to cognitive decline [20]. Together, postmenopausal conditions when paired with obesity may further amplify the risks of cognitive impairment [15,16,21]. Accumulating evidence has demonstrated that postmenopausal women with obesity had poorer cognitive function than premenopausal women [21] and postmenopausal women without obesity [22]. An association between postmenopausal obesity and cognitive impairment, as well as the risk of dementia and Alzheimer’s disease, has been reported in both animal and clinical studies [12,15,16,21,23]. While postmenopausal women with obesity are at increased risk of developing dementia, only few studies have been conducted to identify effective strategies to delay or prevent cognitive impairment in this population.

Physical activity is defined as “any bodily movement produced by skeletal muscles that result in energy expenditure”, whereas the definition of exercise is “a subset of physical activity that is planned, structured and repeatedly done to improve or maintain physical fitness”. However, the dose of physical activity and exercise is similarly described by duration, mode, frequency, and intensity [24]. It is known that both physical activity and exercise play an important role in preventing and reducing risks of obese-related outcomes. In this narrative review, both physical activity and exercise are considered.

Regular physical activity has long been recognized as the primary treatment to prevent obesity and attenuate menopausal-related symptoms [25,26]. Previous studies have reported that the effects of physical exercise on cognitive benefits appear to be stronger in women than in men [27,28]. A growing body of evidence has revealed that regular physical activity was associated with lower rates of cognitive decline in obese participants or participants who underwent postmenopausal condition [26,29]. Among different physical activities and exercise regimens, recent literature has suggested that challenging aging brains through physical–cognitive training might be even more effective against age-related cognitive decline than physical training alone [30,31].

A technology-based intervention has been recently proposed as one of the effective behavioral approaches that could encourage health promotion as well as improve motivational factors in postmenopausal women [32]. There is a growing interest in incorporating physical exercise or physical–cognitive training into a technology-based intervention to improve cognitive function. In this review, we summarize the effects of physical exercise, physical–cognitive training, and technology-based intervention on cognitive performance and the possible mechanisms of these interventions on neurocognitive health in obesity with and without postmenopausal condition.

## 2. Physical Exercise and Cognitive Benefits-Selected Studies

It is well established that physical intervention is an effective method for prevention of cardiometabolic risks, obesity-related problems, as well as menopausal-related symptoms such as depression, anxiety symptoms, and sarcopenia [26,29]. Physical exercise is recommended as an important healthy lifestyle behavior to improve not only musculoskeletal and cardiovascular function but also cognitive performance in middle-aged and older adults [26,29,33,34]. A randomized controlled study demonstrated that moderate-intensity of physical exercise increased hippocampal volume by 2% while the control condition showed a 1.4% reduction in the brain volume of the same area [35]. Findings from a long-term study with a 10-year follow-up suggested the potential benefit of physical exercise in lowering the risk of age-related cognitive impairment [36]. A systematic review with meta-analysis study investigating the effects of physical exercise on cognitive performance in adults older than 50 years revealed that physical exercise including aerobic exercise, resistance training, multicomponent exercise, and Tai chi with a minimum duration of 45 min at moderate to vigorous intensity significantly improved cognitive function regardless of baseline cognitive status [34].

### 2.1. Evidence in Older Adults with Obesity

A few studies have examined the influence of physical fitness on cognitive function in older adults with obesity [37,38,39]. Two cross-sectional studies explored the impact of physical fitness or physical activity on cognitive performance in older adults with obesity [38,39]. Boidin et al. [38] compared cognitive performance among obese individuals (women (30%)) who had high and low cardiorespiratory fitness. Results of this study indicated that higher-fit obese participants had better working memory and executive function than lower-fit obese participants. In another study, Coll-Padrós N et al. [39] compared cognitive performance among obese older adults (women (51%)) who engaged in regular physical activity and no or low physical activity levels. Results showed that participants who engaged in regular physical activity had better global cognition, attention, cognitive flexibility, and working memory than those with no or low physical activity levels. An experimental study by Napoli et al. [37] investigated the effects of physical exercise (multicomponent of exercise: aerobic exercise (65–85% of maximum heart rate (HRmax)), resistance exercise (65–80% of 1 repetition maximum (RM)), balance exercise, 90 min/session, 3 sessions/week for 1 year) on global cognitive function, executive function, and verbal fluency in obese older adults (women (63%)). They found that physical exercise improved global cognitive function and verbal fluency domain; however, improvement in executive function did not reach significance. Collectively, these findings suggest that being fit and engaging in physical exercise have positive effects on cognitive function of obese older adults, which is in agreement with most findings in non-obese older adults.

### 2.2. Evidence in Postmenopausal with Overweight and Obesity

A previous systematic review concluded that physical activity (≥150 min/week) was associated with a lower rate of cognitive impairment in women who underwent postmenopausal condition with and without obesity [26]. De Camargo Smolarek et al. [40] reported that overweight postmenopausal women who engaged in a physical exercise program (resistance exercise (60–70% of 1 RM)) for 12 weeks demonstrated improvement in global cognitive function as measured by the Montreal Cognitive Assessment. As for postmenopausal women with obesity, research studies investigating the effects of physical exercise on cognitive parameters are scarce. To the best of our knowledge, only one study was conducted to investigate such effects. Kim and Kang [25] examined the effects of 12-week physical exercise (aerobic exercise (50% of heart rate reserve (HRR)) and resistance exercise (55–65% of 1 RM)) on physical outcomes, menopausal symptoms, neurotrophic factors, and cognitive function (self-reported cognitive function was determined by the Attention Function Index) in postmenopausal women with obesity. Results showed that physical exercise was effective in improving body composition, alleviating menopausal symptoms, and stimulating neurotrophic factors. However, improvement of cognitive function was not observed. Unlike most studies where cognitive function was objectively evaluated by using neuropsychological tests, in this study, a self-report of perceived effectiveness in daily functioning was used to reflect cognitive ability.

Taken together, while available evidence suggests promising effects of physical exercise on cognitive function of obese individuals, more evidence, especially from randomized clinical trials with a large sample and rigorous methodology, is required for obese individuals with postmenopausal condition. Future research should also examine the interactions between obesity, estrogen deprivation, and physical exercise on cognitive function. Table 1 summarizes clinical studies investigating the effects of physical exercise on cognitive function.

## 3. Associated Physiological Mechanisms of Physical Exercise Induced-Cognitive Improvement in Obese Individuals—Selected Studies

Several mechanisms derived from both animal and human studies have been proposed to explain the positive effects of physical exercise on cognitive function. Among various findings, the associated mechanisms by which physical exercise-induced cognitive improvement could be mainly due to (1) the upregulation of neurotrophic factors, (2) the reduction of insulin resistance, (3) the reduction of pro-inflammatory cytokines, and (4) the increment of anti-inflammatory markers [33,41,42].

Research evidence has demonstrated that the benefits of physical exercise in cognitive performance are mediated through neurotrophic factors (e.g., brain-derived neurotrophic factor (BDNF) and nerve growth factor (NGF)), which are produced following exercise [41,43]. Results from meta-analysis studies indicated that blood concentrations of BDNF are enhanced after exercise interventions [44,45]. Mueller et al. [43] demonstrated that physical exercise improved metabolic dysfunction and altered BDNF concentrations in overweight to obese individuals, all of these related to an increase in brain density. Physical exercise that promotes the synthesis of neurotrophic factors may result in neurogenesis and brain synaptogenesis, thus ameliorating cognitive impairments. In a study conducted by Alizadeh and Dehghanizade [46], physical exercise increased BDNF levels and improved executive function in obese women. Among obese women who underwent postmenopausal conditions, a recent study also demonstrated the positive effects of physical exercise on neurotrophic factors [25]. Results of this study indicated that regular physical activity significantly increased BDNF and NGF levels in postmenopausal women with obesity [25]. In line with findings from the above epidemiological studies, several animal studies have demonstrated that physical exercise stimulates myokines and neurotrophic factors, which enhances cell differentiation, cell survival, dendritic spine growth, synaptic plasticity, and contributes to cognitive improvement.

Another pathological condition that commonly occurs in obese conditions is peripheral insulin resistance, which may contribute to the development of brain insulin resistance and lead to the reduction of cognitive function [47,48]. Previous studies reported that physical exercise is recommended to improve insulin sensitivity and insulin secretion in obese individuals [42,49]. The improvement of insulin sensitivity following exercise training may contribute to cognitive improvement [50,51]. Park et al. [50] demonstrated that physical exercise improved hippocampal insulin signaling, which is accompanied by cognitive improvement in obesity-induced insulin resistance rats. Similarly, Kang and Cho [51] found that physical exercise improved the insulin signaling pathway in rat’s brains along with improved cognitive function. In line with findings from animal studies, a previous study examining the relationships among cognitive function, exercise status, and type 2 diabetes mellitus reported an inverse relationship between insulin resistance and cognitive performance in participants with and without type 2 diabetes mellitus [52]. Together, these findings suggested that improvement in insulin sensitivity may be one mechanism responsible for cognitive improvement after exercise intervention in obese conditions.

Moreover, physical exercise has been shown to decrease levels of pro-inflammatory cytokines (e.g., interleukin 6 (IL-6), tumor necrosis factor alpha (TNF-α), c-reactive protein (CRP)), as well as increase levels of anti-inflammatory cytokines (e.g., adiponectin levels) in obese individuals [53,54,55,56,57,58]. A systemic review and meta-analysis study reported that physical exercise reduced IL-6, decreased leptin, and increased adiponectin levels, corresponding to a reduction of obesity-associated systemic inflammation [53]. Another systemic review and meta-analysis study [54] demonstrated that exercise intervention reduced IL-6, TNF-α, and CRP, and increased adiponectin levels in postmenopausal women. The authors suggested that physical exercise is an effective intervention for lowering pro-inflammatory markers and raising adiponectin levels in women who underwent postmenopausal conditions. Additionally, findings from both animal and human studies demonstrated that physical exercise has an anti-inflammatory effect, which may result in decreased neuroinflammation and enhanced neurogenesis, thus improving cognitive function. Nascimento et al. [58] demonstrated that physical exercise decreased pro-inflammatory markers including TNF-α and IL-6 levels and enhanced neurotrophic factors, along with the improvement in global cognitive function. Similarly, animal studies demonstrated that regular physical activity decreased inflammatory markers and corresponding increases in synaptogenesis and neurogenesis [55,56,57].

**Figure 1:**
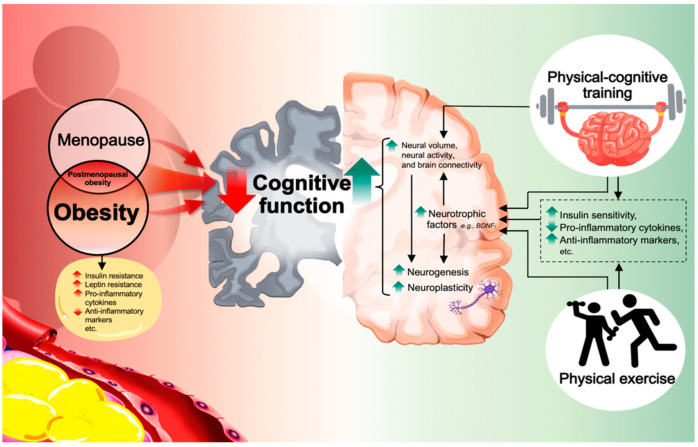
Scheme illustrating the possible mechanisms of physical exercise and physical–cognitive training on cognitive improvement. Obesity and menopause (estrogen deprivation) are both independently associated with cognitive impairment. Further, when overlapping, these conditions may magnify and aggravate cognitive decline. Either obese or menopausal condition is linked with metabolic dysfunction and systematic inflammation, etc., which results in cognitive impairment. Both physical exercise and physical–cognitive training improved insulin sensitivity, decreased pro-inflammatory cytokines, and enhanced anti-inflammatory markers, resulting in improved neurotrophic factors, neurogenesis, and neuroplasticity, and consequently lead to cognitive improvement.

Altogether, available evidence from both animal and human studies demonstrated that physical exercise reduced insulin resistance, decreased pro-inflammatory cytokines, and enhanced anti-inflammatory markers, which result in improved brain insulin signaling, enhanced neurotrophic factors, neurogenesis, angiogenesis, and consequently lead to cognitive improvement. Figure 1 illustrates the possible mechanisms of physical exercise on cognitive improvement.

## 4. Physical–cognitive Intervention and Cognitive Benefits-Selected Studies

Physical–cognitive training (e.g., combined physical–cognitive training, cognitive-motor training, dual-task training, and exergaming) is an intervention that combines physical exercise and cognitive training in a dual-task interference (simultaneous training) or in separate sessions of sequential training [59]. Research evidence has suggested that combined physical and cognitive intervention may provide greater benefits to cognitive performance than single intervention [60,61]. A systematic review by Lauenroth et al. [60] found that eighteen out of twenty studies reported cognitive improvement in the combined physical–cognitive training group. In addition, this study also demonstrated that combined physical–cognitive training has more beneficial effects on cognition compared to single physical or single cognitive training in older adults with and without cognitive impairment [60]. The authors noted that combining aerobic and strength training with attention and/or executive function/working memory appears to be a significant component of an effective training program [60]. Two meta-analysis studies evaluated the potential synergistic effects of combined physical–cognitive intervention in improving cognitive function by comparing cognitive effects following combined interventions to physical intervention, cognitive intervention, and control conditions [30,31]. Both studies concluded that the combined physical–cognitive intervention yielded greater gains in cognitive function of older adults than the control condition and physical intervention, but no significant difference was found between the combined interventions and cognitive intervention alone [30,31].

As for the effects of combined interventions on specific cognitive domains, previous studies demonstrated that they are especially effective in improving memory and executive function [61,62]. The combined physical–cognitive intervention group significantly improved memory when compared to either the cognitive or physical training groups [61]. Although there were no significant differences between the combined intervention and the physical or cognitive intervention alone, this intervention led to significantly larger improvement in executive function when compared to the control group [62].

Inconsistent findings among previous studies are likely due to the heterogeneity of existing studies and the absence of a theoretical framework for analyzing combined interventions [63]. Torre and Temprado [63] proposed a multi-dimensional analysis that considers interactions among seven constructs including stimuli, settings, targets, markers, outcomes, moderators, and mechanisms. By using this framework, the authors concluded that combined training interventions are more effective than separate physical and cognitive training to improve cognitive function in older adults when they are well designed and well conducted [63].

To date, the effects of physical–cognitive training on cognitive performance in obesity with and without postmenopausal condition remains scarce. A study by Staiano et al. [64] demonstrated that 10 weeks of physical–cognitive training (30 min/sessions, ~5 sessions/week) improved executive function in overweight and obese adolescents (women (57%)). Additionally, the results showed that weight loss during the intervention was positively correlated with the improvement of executive function. Garcia-Garro et al. [65] found that 12 weeks of Pilates exercise, a form of mind-body training (60 min/session, 2 sessions/week), significantly improved cognitive and physical abilities of postmenopausal women. The average body mass index of women in their study was 29.4 (SD 4.54) which is considered as overweight. Thus, the findings provided evidence supporting the positive effect of combined physical–cognitive exercise on cognitive function in overweight, postmenopausal women. Another study by Jo et al. [66] demonstrated that 12 weeks of physical–cognitive training (42–82% of HRR, 40 min/session) improved cardiorespiratory fitness and endothelial function in postmenopausal with high cardiovascular risk conditions. Cardiovascular fitness and endothelial function have been suggested as potential mediating factors for cognitive improvement. Nevertheless, cognitive performance was not measured in this study.

In sum, the beneficial effects of combined physical–cognitive training on global cognitive function and specific cognitive domains including memory and executive function have been consistently demonstrated in all age groups, particularly in older adults with and without cognitive impairment. However, there is a limited number of studies that investigated the effects of combined physical–cognitive intervention on cognitive function in obese individuals with and without postmenopausal condition. While accumulating evidence suggests the synergistic effects of the combined physical–cognitive intervention, there is still insufficient evidence to conclude that the combined interventions augment cognitive advantages beyond that of single physical or cognitive intervention. Table 2 summarizes clinical studies investigating the effects of physical–cognitive intervention on cognitive function and health-related outcomes.

## 5. Associated Physiological Mechanisms of Physical and Cognitive Exercise-Induced Cognitive Improvement in Obese Individuals—Selected Studies

Previous studies have demonstrated that physical exercise improved both biochemical and physiological cascades that play an important role in neuroplasticity such as an increase in cerebral blood flow, vascularization, and neurotrophic factors supporting neurogenesis [41,43,67], whereas cognitive training increased brain volume, neural activity, neural connectivity and task-specific activation [68]. Thus, improvement of the neuroplasticity and synaptic integrity in the brain may be boosted when these two interventions are combined. Findings from studies by Anderson-Hanley et al. [69,70] have supported this notion. The authors suggested that the combined training may exert the synergistic effect of physical exercise and cognitive training on cognitive function. Likewise, Bherer et al. [71] examined the synergistic effect of combined physical–cognitive training by comparing its effects with physical training alone, cognitive training alone, or control condition on dual-task performance in older adults. Their findings also supported a synergistic effect of the combined training on cognitive ability. Nevertheless, the potential mechanisms underlying this synergistic effect were not investigated in their study.

Although the underlying mechanisms of combined training-induced cognitive improvement are not well elucidated, emerging evidence has demonstrated that combined physical–cognitive intervention facilitates neurotrophic factors and enhances neuroplasticity [69,72,73]. Among several associated mechanisms of physical–cognitive training on cognitive benefits, the improvement of cognitive performance through the enhancement of BDNF levels has been consistently reported [69,73]. Similarly, studies in animal models reported that combined physical exercise and cognitive stimulation had a complementary effect on neurogenesis [74]. Langdon and Corbett [75] reported that combined physical exercise and cognitive training improved learning and memory and enhanced hippocampal BDNF greater than the physical exercise or cognitive training alone in rat models. A study by Fabel et al. [74] found that combined physical activities and a cognitively challenging environment increased new neurons, which increased neurogenesis in female mice models.

Taken together, the neural mechanisms responsible for the impact of physical–cognitive training on cognitive benefits may be due to the improvement of neural volume, neurotrophic factors, neurogenesis, and synaptic plasticity. Both physical exercise and cognitive training are involved in several brain plasticity mechanisms, supporting the notion that physical–cognitive training could have a synergistic or additive effect on cognition when these two interventions are combined. To better understand the underlying mechanisms of these interventions on cognition, an investigation of biochemical, and physiological outcomes along with neuronal and behavioral changes is needed. Figure 1 illustrates the possible mechanisms of physical–cognitive training on cognitive improvement.

## 6. Technology-Based Interventions for Health and Cognitive Benefits

Although it is well established that exercise promotes health benefits, the uptake and adherence of the exercise programs in all populations including obese individuals are generally low due to a number of reasons including lack of time and motivation [76,77,78]. Recently, technology-based interventions have become a part of healthcare and medical intervention, providing accessible and motivational benefits for health promotion [32,79]. Among various technologies for intervention, telemedicine, exergaming technologies (e.g., Nintendo Wii, Kinect Xbox, and virtual reality systems), mobile technology, and eHealth were commonly used for obesity management by changing behavioral factors that contribute to a healthy lifestyle. Delivering obesity management through technology-based interventions could offer more reach than face-to-face interventions, and they are less time-consuming and more cost-effective for obese individuals. Ozturk and Duruturk [80] found that physical exercise applied through technology-based intervention (45 min/sessions, 3 sessions/week, 6 weeks) during the pandemic of coronavirus disease 2019 (COVID-19) was an effective, safe, and viable approach to improving physical fitness and quality of life in obese individuals (women (51%)). Additionally, previous studies demonstrated that technology-based interventions present a cost-effective and convenient way to decrease body weight and delay the onset of diabetes mellitus [81,82]. Recent meta-analyses demonstrated that engaging in physical exercise through technology-based intervention enhanced physical fitness and induced weight loss in adults with and without obesity [83,84].

There is a growing interest in applying technology-based intervention to improve cognitive outcomes in all age groups [85,86]. Bonnechère et al. [87] demonstrated that cognitive training applied through technology-based intervention (cognitive mobile games: 100 gaming sessions) improved cognitive mobile game scores in all age populations. A review of studies reported that technology-based intervention that combines physical–cognitive exercise with exergame is an enjoyable tool that improves both physical and cognitive function in youth [88] and older adults [89]. Previous studies reported that physical–cognitive exercise applied through technology-based intervention improved global cognitive function, memory, attention, as well as brain function in older adults with and without cognitive impairment [90,91,92]. Furthermore, a systemic review and meta-analysis study has synthesized the available evidence for technology-based interventions for cognitive improvement. A systematic review by Ge et al. [93] evaluated the effects of technology-based cognitive training or rehabilitation interventions to improve cognitive function. The findings from this systematic review showed that technology-based cognitive training or rehabilitation interventions had a significant effect on executive function, attention, memory, as well as global cognitive function, with moderate to large effect sizes. A meta-analysis of the randomized controlled trial study demonstrated that physical–cognitive exercise applied through technology-based intervention improved cognition including executive functions, attention, and visuospatial skills [79]. This study indicated that technology-based intervention with physical–cognitive exercise has moderate to large effect sizes (ES = 0.44) on global cognition. For the executive domain, technology-based intervention with physical–cognitive exercise improved inhibitory control (ES = 0.9) and cognitive flexibility (ES = 0.35) in comparison to the control condition.

Apart from its beneficial effects on cognitive function, the interactive characteristics of exercise with technology-based intervention promote motivation, entertainment, and excitement among the players, resulting in higher exercise adherence than regular exercise [79,94]. Inzitari et al. [32] reported that physical exercise applied through technology-based intervention was appealing to postmenopausal women, which may help improve adherence to exercise. Exercise-based technologies that comprise physical movement, interactivity, social connectivity, or gamification techniques have been shown to promote motivation for exercise and increase adherence to physical intervention [95,96].

In addition, engaging in physical exercise through technology-based intervention is also involved in several biopsychosocial factors such as economic costs and technology usability [97,98]. Therefore, all biopsychosocial aspects should be considered when physical exercise programs are designed. Table 3 summarizes clinical studies investigating the effects of technology-based intervention on cognitive function and health-related outcomes.

## 7. Limitations

This review should be considered in light of some limitations. Given the nature of a narrative review, there are some methodological and interpretation limitations. The selection of studies included in the review might have been unintentionally bias. We acknowledge that obesity is a multifactorial disease involving various biopsychosocial aspects (e.g., physiological, psychological, and social factors). However, these biopsychosocial factors were not within the scope of this review. Currently, only few previous studies investigated the cognitive effects of physical activity or exercise in obese individuals with postmenopausal condition; thus, a narrative review appears appropriate at this stage of research in this population. Another limitation is that both cross-sectional and experimental studies were included in the review due to the limited number of previous studies; thus, evidence of causal link between exercise and cognitive function was compromised. Despite these limitations, this review provides a summary of evidence on the impacts of exercise on cognitive function of obese individuals with postmenopausal condition and suggestion for future research directions.

## 8. Conclusions

This review aimed to summarize the effects of physical exercise, physical–cognitive training, and technology-based intervention on cognitive performance and the potential mechanisms of these interventions on neurocognitive health in obesity, particularly in those with menopausal condition. Despite the scarcity of studies in this population, available evidence has shown promising findings. Clinically, both physical exercise and combined physical–cognitive exercise may potentially be considered as effective non-pharmacological approaches to maintain or improve cognitive function in obese individuals with and without postmenopausal conditions. While the dose (intensity, frequency, duration) of exercise varied across studies, most studies utilized moderate intensity, 30–60 min/session, 3 sessions/week for 12 weeks. Considering the type of exercises, multicomponent exercises comprising aerobic and resistance were often used for physical exercise, while cognitive exercise frequently includes activities that target memory and executive function. Exercise-based technologies have been identified as a promising approach for increasing accessibility and adherence. Nevertheless, these findings should be considered as preliminary; further research investigating the effects of physical exercise, and physical–cognitive training on cognitive performance at both physiological and behavioral levels is required. Further, the types, intensity, and duration of exercise that yield optimal cognitive outcome in obese individuals with menopausal condition should be investigated. Clinical trials with rigorous methodology, large sample, and long-term follow-up are also needed to establish a firm conclusion on the effects of these exercise interventions in delaying or preventing cognitive impairment among obese individuals with and without postmenopausal condition.

## Figures and Tables

**Table 1:** Studies investigating the effects of physical exercise on cognitive function.

Authors	Study Design	Population Characteristics	Methods (Focused on Exercise Intervention and Control)	Cognitive Assessments	Main Results
Evidence in older adults with obesity					
Napoli et al., 2014 [37]	Experimental study (RCT) Groups: Control Exercise Diet Diet-exercise	Gender (women) = 67 (63%) Age = 69.8 ± 4 years BMI = 37.2 ± 5 kg/m^2^	Intervention: exercise training program (Multicomponent of exercise; aerobic exercise (65–85% of HRmax), resistance exercise (65% of 1 RM, 1–2 sets of 8–12 repetitions, gradually increased the intensity to 80% of 1 RM, 2–3 sets of 6–8 repetitions), balance exercise, 90 min/session, 3 sessions/week, 1 year) Control: no exercise	The Modified Mini-Mental State Examination, the Trail Making Test, and the Word List Fluency Test	Exercise group demonstrated better global cognitive function and verbal fluency domain than control group
Boidin et al., 2020 [38]	Cross-sectional study Groups: Higher-fit obese Lower-fit obese Non-obese	Gender (women) = 19 (30%) Age = 62 ± 6 years BMI = 29.7 ± 3.9 kg/m^2^	Participants were classified by their aerobic fitness relative to lean body mass (peak VO_2_/LBM)	Neuropsychological test battery	Higher-fit obese had better working memory and executive function than lower-fit obese participants
Coll-Padrós et al., 2019 [39]	Cross-sectional study Groups: No or low physical activity Regular physical activity	Gender (women) = 42 (51%) Age = 67.1 ± 4.7 years BMI = 31.8 ± 3.0 kg/m^2^	Physical activity was determined by the Rapid Assessment of Physical Activity (RAPA) questionnaire	Neuropsychological test battery	Participants who engaged in regular physical activity had better global cognition, attention, cognitive flexibility, and working memory than no or low physical activity levels
Evidence in postmenopausal with overweight and obesity					
Kim and Kang, 2020 [25]	Experimental study Groups: Premenopausal Postmenopausal	Gender (women) = 52 (100%) Age = 52.68 ± 5.9 years BMI = 25.72 ± 3.6 kg/m^2^	Intervention: exercise training program (Aerobic exercise; 50% of HRR and resistance exercise; 55–65% of 1 RM, 3 sets of 10–12 repetitions, 12 weeks)	The Attention Function Index (self-reported cognitive function)	No significantly improved perceived cognitive functioning in postmenopausal group after intervention
De Camargo Smolarek et al., 2019 [40]	Experimental study Groups: Control Exercise	Gender (women) = 21 (100%) Age = >60 years BMI = 31.05 ± 2.0 kg/m^2^	Intervention: exercise training program (Resistance exercise; 60–70% of 1 RM, 12 weeks) Control: no exercise	The Montreal Cognitive Assessment	Global cognition improvement in exercise group after intervention

Age values and BMI values are mean ± standard deviation. BMI: body mass index; HRmax: maximum heart rate; HRR: heart rate reserve; LBM: lean body mass; RCT: randomized controlled trial; RM: repetition maximum; peak VO_2_: peak oxygen consumption.

**Table 2:** Studies investigating the effects of physical–cognitive intervention on cognitive function and health-related outcomes.

Authors	Study Design	Population Characteristics	Methods (Focused on Physical–Cognitive Intervention and Control)	Cognitive Assessments	Main Results
Evidence in obesity with and without postmenopausal condition					
Staiano et al., 2012 [64]	Experimental study Groups: Control Competitive exergame Cooperative- exergame	Gender (women) = 31 (57%) Age = 16.5 years BMI = 33.1 kg/m^2^	Intervention: physical–cognitive training program (Exergame (the Nintendo Wii EA Sports Active exergame), 30 min/sessions, ~5 sessions/week, 10 weeks) Control: no exercise	The Delis-Kaplan Executive Function System (D-KEFS)	Competitive exergame group demonstrated better executive function than cooperative exergame group and control group
Garcia-Garro et al., 2020 [65]	Experimental study (RCT) Groups: Control Pilates	Gender (women) = 110 (100%) Age = 68.2 ± 8.4 years BMI = 29.4 ± 4.5 kg/m^2^	Intervention: physical–cognitive training program (Pilates exercise (mind-body training), 60 min/session, 2 sessions/week, 12 weeks) Control: no exercise	The Mini-Mental State Examination, Isaacs test, and the Trail Making Test	Pilates group demonstrated better verbal fluency and executive function than control group
Jo et al., 2020 [66]	Experimental study (RCT) Groups: Control Treadmill Exergame	Gender (women) = 65 (100%) Age = 60.5 ± 10.8 years BMI = 27.3 ± 3.5 kg/m^2^	Intervention: physical–cognitive training program (Exergame (the Exer Heart device), 42–82% of HRR, 40 min/session, 12 weeks) Control: no exercise	-	Exergaming improved cardiorespiratory fitness and endothelial function in a similar way to treadmill exercise, but it had better attendance and adherence rates

Age values and BMI values are mean ± standard deviation. BMI: body mass index; HRR: heart rate reserve; RCT: randomized controlled trial.

**Table 3:** Studies investigating the effects of technology-based interventions on cognitive function and health-related outcomes.

Authors	Study Design	Population Characteristics	Methods (Focused on Technology-Based Intervention and Control)	Cognitive Assessments	Main Results
Evidence in obesity, adults, and older adults					
Ozturk et al., 2022 [80]	Experimental study Groups: Control Telerehabilitation	Gender (women) = 21 (51%) Age = 41.0 ± 12.9 years BMI = 30.9 ± 3.0 kg/m^2^	Intervention: telerehabilitation program (trunk stabilization exercises and breathing exercises), 45 min/sessions, 3 sessions/week, 6 weeks Control: no exercise	-	Exercise through telerehabilitation improved physical fitness and quality of life than control group
Toro-Ramos et al., 2017 [82]	Experimental study Groups: Control Intervention	Gender (women) = 46 (29%) Age = 37.4 ± 8.7 years BMI = 28.2 ± 3.4 kg/m^2^	Intervention: smartphone application (the Noom app; lifestyle intervention, 15 weeks) Control: no exercise	-	After intervention, intervention group had lower body weight, lower %fat, and improved metabolic profiles compared to control group
Bonnechère et al., 2020 [87]	Retrospective observational study Groups: 18–24 years old 25–34 years old 35–44 years old 45–55 years old 55–64 years old ≥65 years old	Gender (women) = - Age: 18–24 years = 21.3 ± 2.2 years; 25–34 years = 30.6 ± 3.3 years; 35–44 years = 40.3 ± 4.2 years; 45–54 years = 49.4 ± 3.3 years; 55–64 years = 59.7 ± 3.5 years; ≥65 years = 70.5 ± 4.2 years BMI = -	Cognitive mobile games, 100 gaming sessions	Cognitive mobile game scores	Improved cognitive mobile game scores in all age populations
González-Palau et al., 2014 [90]	Experimental study Groups: Healthy MCI	Gender (women) = 40 (80.5%) Age = 73.4 ± 7.5 years BMI = -	Intervention: computer-based cognitive and physical training program (The Long Lasting Memories program), 60 min/session, 3 sessions/week, 12 weeks	Neuropsychological test battery	Intervention improved global cognition, verbal memory, and episodic memory in both MCI and healthy participants
Styliadis et al., 2015 [91]	Experimental study Groups: Passive control Active control Combined physical–cognitive training Cognitive training Physical training	Gender (women) = 45 (64%) Age = 70.61 ± 5.2 years BMI = -	Combined computerized physical and cognitive training: The Long Lasting Memories program (LLM), 10 h/week, 8 weeks Cognitive training: CT component of LLM, 1 h/session, 5 sessions/week, 8 weeks Physical training: PT component of LLM, 1 h/session, 5 sessions/week, 8 weeks Passive control: no exercise Active control: no exercise, watching documentaries	The Mini-Mental State Examination and electroencephalogram	Improvement of cognitive function was found in combined physical–cognitive group after training
Phirom et al., 2020 [92]	Experimental study Groups: Control Intervention	Gender (women) = 17 (85%) Age = 69.8 ± 3.78 years BMI = -	Intervention: physical–cognitive game-based training (the Xbox Kinect), 60 min/sessions, 3 sessions/week, 12 weeks Control: no exercise	The Montreal Cognitive Assessment	Intervention group demonstrated improvement in global cognitive function than control group

Age values and BMI values are mean ± standard deviation. BMI: body mass index; CT: cognitive training; MCI: mild cognitive impairment; PT: physical training.

## References

[1] Malenfant J.H., Batsis J.A. (2019). Obesity in the geriatric population—A global health perspective. J. Glob. Health Rep..

[2] Ng M., Fleming T., Robinson M., Thomson B., Graetz N., Margono C., Mullany E.C., Biryukov S., Abbafati C., Abera S.F. (2014). Global, regional, and national prevalence of overweight and obesity in children and adults during 1980–2013: A systematic analysis for the Global Burden of Disease Study 2013. Lancet.

[3] Lee A., Cardel M., Donahoo W.T., Feingold K.R., Anawalt B., Boyce A., Chrousos G., de Herder W.W., Dhatariya K., Dungan K. (2000). Social and Environmental Factors Influencing Obesity.

[4] Zatońska K., Psikus P., Basiak-Rasała A., Stępnicka Z., Gaweł-Dąbrowska D., Wołyniec M., Gibka J., Szuba A., Połtyn-Zaradna K. (2021). Obesity and Chosen Non-Communicable Diseases in PURE Poland Cohort Study. Int. J. Environ. Res. Public Health.

[5] Villareal D.T., Apovian C.M., Kushner R.F., Klein S. (2005). Obesity in older adults: Technical review and position statement of the American Society for Nutrition and NAASO, The Obesity Society. Am. J. Clin. Nutr..

[6] Pasquali R., Casanueva F., Haluzik M., van Hulsteijn L., Ledoux S., Monteiro M.P., Salvador J., Santini F., Toplak H., Dekkers O.M. (2020). European Society of Endocrinology Clinical Practice Guideline: Endocrine work-up in obesity. Eur. J. Endocrinol..

[7] Whitmer A., Gunderson P., Barrett-Connor E., Quesenberry J., Yaffe K. (2005). Obesity in middle age and future risk of dementia: A 27 year longitudinal population based study. BMJ (Clin. Res.).

[8] Kivipelto M., Ngandu T., Fratiglioni L., Viitanen M., Kareholt I., Winblad B., Helkala E.L., Tuomilehto J., Soininen H., Nissinen A. (2005). Obesity and vascular risk factors at midlife and the risk of dementia and Alzheimer disease. Arch. Neurol..

[9] Whitmer R.A., Gunderson E.P., Quesenberry C.P., Zhou J., Yaffe K. (2007). Body mass index in midlife and risk of Alzheimer disease and vascular dementia. Curr. Alzheimer Res..

[10] Beam R., Kaneshiro C., Jang Y., Reynolds C.A., Pedersen N.L., Gatz M. (2018). Differences between women and men in incidence rates of dementia and alzheimer’s disease. J. Alzheimers Dis..

[11] Chêne G., Beiser A., Au R., Preis S.R., Wolf P.A., Dufouil C., Seshadri S. (2015). Gender and incidence of dementia in the Framingham Heart Study from mid-adult life. Alzheimers Dement..

[12] De Franciscis P., Barbieri M., Leo S., Dalise A.M., Sardu C., Marfella R., Colacurci N., Paolisso G., Rizzo M.R. (2017). Serum adiponectin levels are associated with worse cognitive function in postmenopausal women. PLoS ONE.

[13] Kaur S., Gonzales M.M., Tarumi T., Villalpando A., Alkatan M., Pyron M., Tanaka H., Haley A.P. (2016). Serum brain-derived neurotrophic factor mediates the relationship between abdominal adiposity and executive function in middle age. J. Int. Neuropsychol. Soc..

[14] Benito-León J., Mitchell A.J., Hernández-Gallego J., Bermejo-Pareja F. (2013). Obesity and impaired cognitive functioning in the elderly: A population-based cross-sectional study (NEDICES). Eur. J. Neurol..

[15] Pratchayasakul W., Sa-Nguanmoo P., Sivasinprasasn S., Pintana H., Tawinvisan R., Sripetchwandee J., Kumfu S., Chattipakorn N., Chattipakorn S.C. (2015). Obesity accelerates cognitive decline by aggravating mitochondrial dysfunction, insulin resistance and synaptic dysfunction under estrogen-deprived conditions. Horm. Behav..

[16] Christensen A., Pike C.J. (2015). Menopause, obesity and inflammation: Interactive risk factors for Alzheimer’s disease. Front. Aging Neurosci..

[17] Forny-Germano L., De Felice F.G., Vieira M. (2018). The Role of Leptin and Adiponectin in Obesity-Associated Cognitive Decline and Alzheimer’s Disease. Front. Neurosci..

[18] Kivimäki M., Luukkonen R., Batty G.D., Ferrie J.E., Pentti J., Nyberg S.T., Shipley M.J., Alfredsson L., Fransson E.I., Goldberg M. (2018). Body mass index and risk of dementia: Analysis of individual-level data from 1.3 million individuals. Alzheimers Dement..

[19] Weber M.T., Maki P.M., McDermott M.P. (2014). Cognition and mood in perimenopause: A systematic review and meta-analysis. J. Steroid Biochem. Mol. Biol..

[20] Hara Y., Waters E.M., McEwen B.S., Morrison J.H. (2015). Estrogen effects on cognitive and synaptic health over the lifecourse. Physiol. Rev..

[21] Parvatha N., Neelambikai N. (2018). Effect of body mass index and waist hip ratio on cognitive performance in pre- and post-menopausal women. Indian J. Appl. Res..

[22] Kumar N. (2014). Effect of abdominal obesity and serum lipid markers on cognitive functions in postmenopausal indian women. World J. Pharm. Pharm. Sci..

[23] Feinkohl I., Lachmann G., Brockhaus W.R., Borchers F., Piper S.K., Ottens T.H., Nathoe H.M., Sauer A.M., Dieleman J.M., Radtke F.M. (2018). Association of obesity, diabetes and hypertension with cognitive impairment in older age. Clin. Epidemiol..

[24] Chang Y.K., Chu C.H., Chen F.T., Hung T.M., Etnier J.L. (2017). Combined Effects of Physical Activity and Obesity on Cognitive Function: Independent, Overlapping, Moderator, and Mediator Models. Sport. Med..

[25] Kim B., Kang S. (2020). Regular leisure-time physical activity is effective in boosting neurotrophic factors and alleviating menopause symptoms. Int. J. Environ. Res. Public Health.

[26] Anderson D., Seib C., Rasmussen L. (2014). Can physical activity prevent physical and cognitive decline in postmenopausal women? A systematic review of the literature. Maturitas.

[27] Barha C.K., Davis J.C., Falck R.S., Nagamatsu L.S., Liu-Ambrose T. (2017). Sex differences in exercise efficacy to improve cognition: A systematic review and meta-analysis of randomized controlled trials in older humans. Front. Neuroendocrinol..

[28] Colcombe S., Kramer A.F. (2003). Fitness effects on the cognitive function of older adults: A meta-analytic study. Psychol. Sci..

[29] Baker A., Sirois-Leclerc H., Tulloch H. (2016). The impact of long-term physical activity interventions for overweight/obese postmenopausal women on adiposity indicators, physical capacity, and mental health outcomes: A systematic review. J. Obes..

[30] Zhu X., Yin S., Lang M., He R., Li J. (2016). The more the better? A meta-analysis on effects of combined cognitive and physical intervention on cognition in healthy older adults. Ageing Res. Rev..

[31] Gheysen F., Poppe L., DeSmet A., Swinnen S., Cardon G., De Bourdeaudhuij I., Chastin S., Fias W. (2018). Physical activity to improve cognition in older adults: Can physical activity programs enriched with cognitive challenges enhance the effects? A systematic review and meta-analysis. Int. J. Behav. Nutr. Phys. Act..

[32] Inzitari M., Greenlee A., Hess R., Perera S., Studenski S.A. (2009). Attitudes of postmenopausal women toward interactive video dance for exercise. J. Womens Health (Larchmt).

[33] Wang C., Chan Y., Ren L., Yan H. (2016). Obesity reduces cognitive and motor functions across the lifespan. Neural Plast..

[34] Northey J., Cherbuin N., Pumpa K., Smee D., Rattray B. (2017). Exercise interventions for cognitive function in adults older than 50: A systematic review with meta-analysis. Br. J. Sport. Med..

[35] Erickson K., Voss M., Prakash R., Basak C., Szabo-Reed A., Chaddock L., Kim J., Heo S., Alves H., Phillips S. (2011). Exercise training increases size of hippocampus and improves memory. Proc. Natl. Acad. Sci. USA.

[36] Jedrziewski K., Ewbank C., Wang H., Trojanowski Q. (2010). Exercise and cognition: Results from the National Long Term Care Survey. Alzheimers Dement..

[37] Napoli N., Shah K., Waters D.L., Sinacore D.R., Qualls C., Villareal D.T. (2014). Effect of weight loss, exercise, or both on cognition and quality of life in obese older adults. Am. J. Clin. Nutr..

[38] Boidin M., Handfield N., Ribeiro P., Desjardins-Crépeau L., Gagnon C., Lapierre G., Gremeaux V., Lalongé J., Nigam A., Juneau M. (2020). Obese but Fit: The benefits of fitness on cognition in obese older adults. Can. J. Cardiol..

[39] Coll-Padrós N., León M., Valech N., Ros E., Vidal J., Estruch R., Fitó M., Salas-Salvadó J., Corella D., Molinuevo J.L. (2019). Physical activity is associated with better global cognition and frontal function in overweight/obese older adults with metabolic syndrome. Eur. Rev. Aging Phys. Act..

[40] De Camargo Smolarek A., Boiko Ferreira L.H., Schoenfeld B., Ribeiro Cordeiro G., Alessi A., Laat E., Mascarenhas L., Carvalho Perin S., Zandoná B., Souza W. (2019). Cognitive performance changes after a 12-week strength training program in overweight older women. J. Exerc. Physiol. Online.

[41] Dominguez-Sanchez A., Bustos-Cruz H., Velasco-Orjuela P., Quintero P., Tordecilla-Sanders A., Correa-Bautista E., Triana-Reina R., Garcia-Hermoso A., Gonzalez-Ruiz K., Pena-Guzman A. (2018). Acute effects of high intensity, resistance, or combined protocol on the increase of level of neurotrophic factors in physically inactive overweight adults: The BrainFit Study. Front. Physiol..

[42] Khoo J., Dhamodaran S., Chen D., Yap Y., Chen Y., Tian H. (2015). Exercise-induced weight loss is more effective than dieting for improving adipokine profile, insulin resistance, and inflammation in obese men. Int. J. Sport Nutr. Exerc. Metab..

[43] Mueller K., Moller E., Horstmann A., Busse F., Lepsien J., Bluher M., Stumvoll M., Villringer A., Pleger B. (2015). Physical exercise in overweight to obese individuals induces metabolic- and neurotrophic-related structural brain plasticity. Front. Hum. Neurosci..

[44] Dinoff A., Herrmann N., Swardfager W., Lanctôt K.L. (2017). The effect of acute exercise on blood concentrations of brain-derived neurotrophic factor in healthy adults: A meta-analysis. Eur. J. Neurosci..

[45] Feter N., Alt R., Dias M.G., Rombaldi A.J. (2019). How do different physical exercise parameters modulate brain-derived neurotrophic factor in healthy and non-healthy adults? A systematic review, meta-analysis and meta-regression. Sci. Sports.

[46] Alizadeh M., Dehghanizade J. (2022). The effect of functional training on level of brain-derived neurotrophic factor and functional performance in women with obesity. Physiol. Behav..

[47] Pratchayasakul W., Kerdphoo S., Petsophonsakul P., Pongchaidecha A., Chattipakorn N., Chattipakorn S.C. (2011). Effects of high-fat diet on insulin receptor function in rat hippocampus and the level of neuronal corticosterone. Life Sci..

[48] Sripetchwandee J., Chattipakorn N., Chattipakorn S.C. (2018). Links between obesity-induced brain insulin resistance, brain mitochondrial dysfunction, and dementia. Front. Endocrinol..

[49] Shih K.-C., Kwok C. (2018). Exercise reduces body fat and improves insulin sensitivity and pancreatic β-cell function in overweight and obese male Taiwanese adolescents. BMC Pediatr..

[50] Park H.S., Park S.S., Kim C.J., Shin M.S., Kim T.W. (2019). Exercise alleviates cognitive functions by enhancing hippocampal insulin signaling and neuroplasticity in high-fat diet-induced obesity. Nutrients.

[51] Kang E.B., Cho J.Y. (2014). Effects of treadmill exercise on brain insulin signaling and β-amyloid in intracerebroventricular streptozotocin induced-memory impairment in rats. J. Exerc. Nutr. Biochem..

[52] Colberg S.R., Somma C.T., Sechrist S.R. (2008). Physical activity participation may offset some of the negative impact of diabetes on cognitive function. J. Am. Med. Dir. Assoc..

[53] Sirico F., Bianco A., D’Alicandro G., Castaldo C., Montagnani S., Spera R., Di Meglio F., Nurzynska D. (2018). Effects of physical exercise on adiponectin, leptin, and inflammatory markers in childhood obesity: Systematic review and meta-analysis. Child. Obes..

[54] Khalafi M., Malandish A., Rosenkranz S.K. (2021). The impact of exercise training on inflammatory markers in postmenopausal women: A systemic review and meta-analysis. Exp. Gerontol..

[55] Casaletto K.B., Lindbergh C.A., VandeBunte A., Neuhaus J., Schneider J.A., Buchman A.S., Honer W.G., Bennett D.A. (2022). Microglial correlates of late life physical activity: Relationship with synaptic and cognitive aging in older adults. J. Neurosci..

[56] Nichol K., Poon W., Parachikova A., Cribbs D., Glabe C., Cotman C. (2008). Exercise alters the immune profile in Tg2576 Alzheimer mice toward a response coincident with improved cognitive performance and decreased amyloid. J. Neuroinflamm..

[57] Svensson M., Lexell J., Deierborg T. (2015). Effects of physical exercise on neuroinflammation, neuroplasticity, neurodegeneration, and behavior: What We Can Learn From Animal Models in Clinical Settings. Neurorehabil. Neural Repair.

[58] Nascimento C.M., Pereira J.R., de Andrade L.P., Garuffi M., Talib L.L., Forlenza O.V., Cancela J.M., Cominetti M.R., Stella F. (2014). Physical exercise in MCI elderly promotes reduction of pro-inflammatory cytokines and improvements on cognition and BDNF peripheral levels. Curr. Alzheimer Res..

[59] Gavelin H.M., Dong C., Minkov R., Bahar-Fuchs A., Ellis K.A., Lautenschlager N.T., Mellow M.L., Wade A.T., Smith A.E., Finke C. (2021). Combined physical and cognitive training for older adults with and without cognitive impairment: A systematic review and network meta-analysis of randomized controlled trials. Ageing Res. Rev..

[60] Lauenroth A., Ioannidis A.E., Teichmann B. (2016). Influence of combined physical and cognitive training on cognition: A systematic review. BMC Geriatr..

[61] Fabre C., Chamari K., Mucci P., Massé-Biron J., Préfaut C. (2002). Improvement of cognitive function by mental and/or individualized aerobic training in healthy elderly subjects. Int. J. Sport. Med..

[62] Guo W., Zang M., Klich S., Kawczyński A., Smoter M., Wang B. (2020). Effect of combined physical and cognitive interventions on executive functions in OLDER Adults: A meta-analysis of outcomes. Int. J. Environ. Res. Public Health.

[63] Torre M.M., Temprado J.J. (2021). A review of combined training studies in older adults according to a new categorization of conventional interventions. Front. Aging Neurosci..

[64] Staiano A.E., Abraham A.A., Calvert S.L. (2012). Competitive versus cooperative exergame play for African American adolescents’ executive function skills: Short-term effects in a long-term training intervention. Dev. Psychol..

[65] García-Garro P.A., Hita-Contreras F., Martínez-Amat A., Achalandabaso-Ochoa A., Jiménez-García J.D., Cruz-Díaz D., Aibar-Almazán A. (2020). Effectiveness of a pilates training program on cognitive and functional abilities in postmenopausal women. Int. J. Environ. Res. Public Health.

[66] Jo E.-A., Wu S.-S., Han H.-R., Park J.-J., Park S., Cho K.-I. (2020). Effects of exergaming in postmenopausal women with high cardiovascular risk: A randomized controlled trial. Clin. Cardiol..

[67] Barnes J.N., Corkery A.T. (2018). Exercise improves vascular function, but does this translate to the brain?. Brain Plast..

[68] van Balkom T.D., van den Heuvel O.A., Berendse H.W., van der Werf Y.D., Vriend C. (2020). The effects of cognitive training on brain network activity and connectivity in aging and neurodegenerative diseases: A systematic review. Neuropsychol. Rev..

[69] Anderson-Hanley C., Arciero P.J., Brickman A.M., Nimon J.P., Okuma N., Westen S.C., Merz M.E., Pence B.D., Woods J.A., Kramer A.F. (2012). Exergaming and older adult cognition: A cluster randomized clinical trial. Am. J. Prev. Med..

[70] Anderson-Hanley C., Barcelos N.M., Zimmerman E.A., Gillen R.W., Dunnam M., Cohen B.D., Yerokhin V., Miller K.E., Hayes D.J., Arciero P.J. (2018). The aerobic and cognitive exercise study (ACES) for community-dwelling older adults with or at-risk for mild cognitive impairment (MCI): Neuropsychological, neurobiological and neuroimaging outcomes of a randomized clinical trial. Front. Aging Neurosci..

[71] Bherer L., Gagnon C., Langeard A., Lussier M., Desjardins-Crépeau L., Berryman N., Bosquet L., Vu T.T.M., Fraser S., Li K.Z.H. (2021). Synergistic effects of cognitive training and physical exercise on dual-task performance in older adults. J. Gerontol. B Psychol. Sci. Soc. Sci..

[72] Anderson-Hanley C., Stark J., Wall K.M., VanBrakle M., Michel M., Maloney M., Barcelos N., Striegnitz K., Cohen B.D., Kramer A.F. (2018). The interactive Physical and Cognitive Exercise System (iPACES™): Effects of a 3-month in-home pilot clinical trial for mild cognitive impairment and caregivers. Clin. Interv. Aging.

[73] Rahe J., Becker J., Fink G.R., Kessler J., Kukolja J., Rahn A., Rosen J.B., Szabados F., Wirth B., Kalbe E. (2015). Cognitive training with and without additional physical activity in healthy older adults: Cognitive effects, neurobiological mechanisms, and prediction of training success. Front. Aging Neurosci..

[74] Fabel K., Wolf S.A., Ehninger D., Babu H., Leal-Galicia P., Kempermann G. (2009). Additive effects of physical exercise and environmental enrichment on adult hippocampal neurogenesis in mice. Front. Neurosci..

[75] Langdon K.D., Corbett D. (2012). Improved working memory following novel combinations of physical and cognitive activity. Neurorehabil. Neural Repair.

[76] Collado-Mateo D., Lavín-Pérez A.M., Peñacoba C., Del Coso J., Leyton-Román M., Luque-Casado A., Gasque P., Fernández-Del-Olmo M., Amado-Alonso D. (2021). Key Factors Associated with Adherence to Physical Exercise in Patients with Chronic Diseases and Older Adults: An Umbrella Review. Int. J. Environ. Res. Public Health.

[77] Othman M.S., Mat Ludin A.F., Chen L.L., Hossain H., Abdul H., Sameeha M.J., Tahir A.R.M. (2022). Motivations, barriers and exercise preferences among female undergraduates: A need assessment analysis. PLoS ONE.

[78] Baillot A., Chenail S., Barros Polita N., Simoneau M., Libourel M., Nazon E., Riesco E., Bond D.S., Romain A.J. (2021). Physical activity motives, barriers, and preferences in people with obesity: A systematic review. PLoS ONE.

[79] Stanmore E., Stubbs B., Vancampfort D., de Bruin E.D., Firth J. (2017). The effect of active video games on cognitive functioning in clinical and non-clinical populations: A meta-analysis of randomized controlled trials. Neurosci. Biobehav. Rev..

[80] Ozturk B., Duruturk N. (2022). Effect of telerehabilitation applied during COVID-19 isolation period on physical fitness and quality of life in overweight and obese individuals. Int. J. Obes..

[81] Cai X., Qiu S., Luo D., Wang L., Lu Y., Li M. (2020). Mobile Application Interventions and Weight Loss in Type 2 Diabetes: A Meta-Analysis. Obesity.

[82] Toro-Ramos T., Lee D.H., Kim Y., Michaelides A., Oh T.J., Kim K.M., Jang H.C., Lim S. (2017). Effectiveness of a Smartphone Application for the Management of Metabolic Syndrome Components Focusing on Weight Loss: A Preliminary Study. Metab. Syndr. Relat. Disord..

[83] Romeo A., Edney S., Plotnikoff R., Curtis R., Ryan J., Sanders I., Crozier A., Maher C. (2019). Can smartphone apps increase physical activity? Systematic review and meta-analysis. J. Med. Internet Res..

[84] Beleigoli A.M., Andrade A.Q., Cançado A.G., Paulo M.N., Diniz M.D.F.H., Ribeiro A.L. (2019). Web-based digital health interventions for weight loss and lifestyle habit changes in overweight and obese adults: Systematic review and meta-analysis. J. Med. Internet Res..

[85] Di Lorito C., Bosco A., Rai H., Craven M., McNally D., Todd C., Booth V., Cowley A., Howe L., Harwood R.H. (2022). A systematic literature review and meta-analysis on digital health interventions for people living with dementia and Mild Cognitive Impairment. Int. J. Geriatr. Psychiatry.

[86] Cacciante L., Pietà C.D., Rutkowski S., Cieślik B., Szczepańska-Gieracha J., Agostini M., Kiper P. (2022). Cognitive telerehabilitation in neurological patients: Systematic review and meta-analysis. Neurol. Sci..

[87] Bonnechère B., Bier J.-C., Van Hove O., Sheldon S., Samadoulougou S., Kirakoya-Samadoulougou F., Klass M. (2020). Age-associated capacity to progress when playing cognitive mobile games: Ecological retrospective observational study. JMIR Serious Games.

[88] Best J.R. (2013). Exergaming in youth: Effects on physical and cognitive health. Z. Psychol..

[89] Bleakley C.M., Charles D., Porter-Armstrong A., McNeill M.D., McDonough S.M., McCormack B. (2015). Gaming for health: A systematic review of the physical and cognitive effects of interactive computer games in older adults. J. Appl. Gerontol..

[90] González-Palau F., Franco M., Bamidis P., Losada R., Parra E., Papageorgiou S.G., Vivas A.B. (2014). The effects of a computer-based cognitive and physical training program in a healthy and mildly cognitive impaired aging sample. Aging Ment. Health.

[91] Styliadis C., Kartsidis P., Paraskevopoulos E., Ioannides A.A., Bamidis P.D. (2015). Neuroplastic effects of combined computerized physical and cognitive training in elderly individuals at risk for dementia: An eLORETA Controlled Study on Resting States. Neural Plast..

[92] Phirom K., Kamnardsiri T., Sungkarat S. (2020). Beneficial effects of interactive physical-cognitive game-based training on fall risk and cognitive performance of older adults. Int. J. Environ. Res. Public Health.

[93] Ge S., Zhu Z., Wu B., McConnell E.S. (2018). Technology-based cognitive training and rehabilitation interventions for individuals with mild cognitive impairment: A systematic review. BMC Geriatr..

[94] Wu S., Jo E.-A., Ji H., Kim K.-H., Park J.-J., Kim B.H., Cho K.I. (2019). Exergaming improves executive functions in patients with metabolic syndrome: Randomized controlled trial. JMIR Serious Games.

[95] De Croon R., Geuens J., Verbert K., Vanden Abeele V. A systematic review of the effect of gamification on adherence across disciplines. Proceedings of the HCI in Games: Experience Design and Game Mechanics: Third International Conference.

[96] Baranowski T., Buday R., Thompson D.I., Baranowski J. (2008). Playing for real: Video games and stories for health-related behavior change. Am. J. Prev. Med..

[97] Valenzuela T., Okubo Y., Woodbury A., Lord S.R., Delbaere K. (2018). Adherence to Technology-Based Exercise Programs in Older Adults: A Systematic Review. J. Geriatr. Phys. Ther..

[98] Campelo A.M., Katz L. (2020). Older Adults’ Perceptions of the Usefulness of Technologies for Engaging in Physical Activity: Using Focus Groups to Explore Physical Literacy. Int. J. Environ. Res. Public Health.

